# Supplementary material for: Sleep-like unsupervised replay reduces catastrophic forgetting in artificial neural networks
Source: Nat Commun. 2022 Dec 15;13:7742. doi: 10.1038/s41467-022-34938-7 (PMC9755223; doi:10.1038/s41467-022-34938-7)
Supplement: Supplementary file 1 — Supplementary Information [file 41467_2022_34938_MOESM1_ESM.pdf]

Supplementary Information for "Sleep-like Unsupervised Replay Reduces Catastrophic Forgetting in Artificial Neural Networks".

Timothy Tadros, Giri P. Krishnan, Ramyaa Ramyaa, Maxim Bazhenov

1. Analysis of similarity of network weights in continual learning scenario

To support our statement that the information about previous tasks is not completely erased after new training, here we examine the cosine similarity between network parameters before and after new task training for two classification tasks trained sequentially (T_1 and T_2). We show that the cosine similarity or the dot product of normalized weight vectors that correspond to the network weights before and after training on new task is positive. We first provide lemmas on vector dot products, which then are applied for analysis of toy model.

Lemmas. The following properties of vector dot products will be used in subsequent sections.

Lemma 1. *Let $\vec{U}$ and $\vec{J}$ be two vectors. The maximum value of $\Sigma_i U_i * J_i = ||\vec{U}|| * ||\vec{J}||$.*

Proof. Since $\Sigma_i U_i * J_i \vec{U} \cdot \vec{J} = ||\vec{U}|| * ||\vec{J}|| \cos(\theta)$ where θ is the angle between $\vec{U}$ and $\vec{J}$, the maximum value of $\Sigma_i U_i * J_i = ||\vec{U}|| * ||\vec{J}||$. □

Lemma 2. *Let $\vec{U}$ and $\vec{J}$ be vectors of length n such that $\vec{U} \cdot \vec{J} > 0$. Let $\vec{V}$ be $\vec{U} - \alpha\vec{J}$ for some α such that $\vec{V} \cdot \vec{J} = 0$. Then $\vec{U} \cdot \vec{V} \geq 0$, with $\vec{U} \cdot \vec{V} = 0$ only when the angle between $\vec{U}$ and $\vec{J}$ is 0.*

Proof. $\vec{U} \cdot \vec{V} = \Sigma_i U_i * (U_i - \alpha * J_i)$.
$= (||\vec{U}||)^2 - \alpha \Sigma_i U_i * J_i$.
Minimizing this expression involves maximizing α and $\Sigma_i U_i * J_i$. By lemma 1, the maximum value of $\Sigma_i U_i * J_i$ is $||\vec{U}|| * ||\vec{J}||$.

To compute the maximum value of α: We know $\vec{V} \cdot \vec{J} = 0$, i.e., $(\vec{U} - \alpha\vec{J}) \cdot \vec{J} = 0$. So, $\Sigma_i U_i * J_i - \alpha \Sigma_i J_i^2 = 0$ So, $\alpha = \vec{U} \cdot \vec{J} / \Sigma_i J_i^2$. Since $\vec{U} \cdot \vec{J} = ||\vec{U}|| * ||\vec{J}|| * cos(\theta)$ where θ is the angle between $\vec{U}$ and $\vec{J}$, the maximum value of α is $||\vec{U}|| * ||\vec{J}|| / \Sigma_i J_i^2$, i.e., $||\vec{U}|| / ||\vec{J}||$.

So, the minimum value of $\vec{U} \cdot \vec{V}$ is $(||\vec{U}||)^2 - ||\vec{U}|| / ||\vec{J}|| * ||\vec{U}|| * ||\vec{J}||$, i.e., 0. Since the maximum values of α and $\Sigma_i U_i * J_i$ occurs when the angle between $\vec{U}$ and $\vec{J}$ is 0, any other situations makes $\vec{U} \cdot \vec{V}$ positive. □

Toy Model Problem Setting. The data set has f features and four possible labels - L_1, L_2, L_3 and L_4. Task 1 (T_1) consists of separating instances of L_1 and L_2 (tested only on these instances). T_2 consists of separating instances of L_3 and L_4 (tested only on these instances). A neural network N can be trained to classify the data: N has f inputs and 4 output neurons O_1, O_2, O_3 and O_4, each one intended to indicate a unique label, i.e., O_i is intended to be true iff the input instance has label i. The training of N happens in 2 phases. In phase 1, N is trained to perform T_1, i.e., the training set consists only of instances labeled 1 or 2. (So, neurons O_3 and O_4 are trained to output only 0's. In phase 2 N is trained to perform T_2, i.e., the training set consists only of instances labeled 3 or 4. (neurons O_3 or O_4 are trained to output 1's for the correct inputs, and O_1 and O_2 are set to 0's for these inputs). After phase 1 and phase 2 training, N performs correctly on T_2, but may be incorrect on T_1, i.e. forget phase 1 training. This happens when on inputs corresponding to category 1 or 2, they output 0, since T_2 always sets O_1 and O_2 to 0. Here, we show that when this is the case, the network has not entirely forgotten phase$_1$ training. for Relu activation and binary inputs.

Perceptron. Here, we consider N to be a perceptron, i.e., inputs are directly connected to output neurons.

After phase 1, let O_1 be the perceptron with $g = f + 1$ weights given by $\vec{W}$ and bias b with activation RELU, i.e., i.e., $O_1(I) = \text{RELU}(\Sigma_{i \leq f} I_i * w_i)$. ($W_g$ is the bias, and the last input is set to 1). So, if $\vec{I}$ with label 1 is input, then $(I \cdot w) > 0$.

Let phase$_2$ training involve an input J, say of label 3, so the desired outputs of neurons O_1, O_2, O_3 and O_4 are $0, 0, 1, 0$. Further, let O_1 on input J output $D > 0$, which is a misclassification and the weights of O_1 would be updated to fix this, i.e., make the output be 0. With perceptron learning algorithm or gradient descent, this would mean $\vec{W}' = \vec{W} - \alpha * \vec{J}$ where α is the learning rate.

By Lemma 2, the cosine similarity between $\vec{W}$ and $\vec{W'}$ is non-negative (and is 0 for just one weight vector). By symmetry arguments, an arbitrary weight vector V would has an expected cosine similarity is zero, we can conclude that the phase 1 training is not fully forgotten after phase 2 training.

Multilayer Perceptron (MPL). Here, we consider N to be two layers of a perceptron (the argument can be extended to multi layers) - a hidden layer H and an output layer O with 4 output neurons. Let U be the set of weights from input to hidden layer, i.e., $U_{i,j}$ is the weight from i^{th} input to the jth hidden neuron. Let V be the set of weights from hidden to output layer, i.e., $V_{i,j}$ is the weight from i^{th} hidden neuron to the jth output neuron. Further, let all the activations be Relu. Using standard backpropogation, the weight update for $V_{i,j}$ is proportional to h_i, and argument for cosine similarity of V and updated V being non-negative is exactly as for the case of the perceptron. For the weights leading to the hidden neuron H_j, the weight update would be proportional to input i (for $U_{i,j}$) and also proportional to $V_{j,1}$ (assuming the setting as before where an error was made on output 1, and only output 1). Since this value is constant for the update over all the weights leading to the hidden neuron H_j, the argument need not change.

2. Analysis of catastrophic forgetting and sleep in toy model.

In this section, we examine the cause of the catastrophic failure and the role of sleep in recovering from the forgetting in toy model. While this example is not intended to model all scenarios of catastrophic forgetting, it provides the intuition and explains how our algorithm prevents catastrophic forgetting.

Let us consider the 3-layer network trained on two categories, each with just one example. Consider 2 binary vectors (Category 1 and Category 2) with some region of overlap. We consider ReLU activation since it was used in the rest of this work. We assume the output to be the neuron with the highest activation in the output layer. Let the network be trained on Category 1 with backpropagation using static learning rate. Following this, we trained the network on Category 2 using same approach. A 3-layer network we consider here has an input layer with 10 neurons, 30 hidden neuron and an output layer with 2 neurons for the 2 categories. Inputs are 10 bits long with 5 bit overlap. We trained with learning rate of 0.1 for 4 epochs.

Analysis of hidden layer behaviour: We can divide the hidden neurons into four types based on their activation for the two categories: A - those neurons that fire for Category 1 but not 2; B - those neurons that fire for Category 2 but not 1; C - those neurons that fire for Category 1 and 2; D - those that fire for neither category, where firing indicates a non-zero activation. Note that these sets may change during training or sleep. Let X_i be the weights from type X to output i.

Consider the case where the input of Category 1 is presented. The only hidden layer neurons that fire are A and C. Output neuron 1 will get the net value $A * A_1 + C * C_1$ and output neuron 2 will get the net value $A * A_2 + C * C_2$. For output neuron 1 to fire, we need two conditions to be held: (1) $A * A_1 + C * C_1 > 0$ (2) $A * A_1 + C * C_1 > A * A_2 + C * C_2$. The second condition above can be rewritten as $A * A_2 - A * A_1 < C * C_1 - C * C_2$, which separates the weights according to the hidden neurons. Using this separation, we give the following definitions: Define a to be $(A_2 - A_1) * A$ on pattern 1; b to be $(A_2 - A_1) * A$ on pattern 2; p to be $(C_1 - C_2) * C$ on pattern 1 and q to be $(C_1 - C_2) * C$ on pattern 2. (Note that p and q are very closely correlated since they differ only in the activation values of C neurons which are positive in both cases).

So, on the input pattern 1, output 1 fires only if $a < p$; on input pattern 2, output 2 fires only if $q < b$.

Catastrophic forgetting: Following training on 2 categories, if the network can not recall Category 1, i.e., output neuron 1 activation is negative or less than that of output neuron 2, catastrophic forgetting has occurred (We confirmed this occurred 78% of times for the 3 layer network described above and 100 trials). The second phase of training ensures $q < b$. This could involve reduction in q which would reduce p as well. (Since A does not fire on input pattern 2, back-propagation does not alter a) Reducing p may result in failing the condition $a < p$, i.e., misclassifying input 1.

Effect of sleep: Sleep can increase difference among weights (which are different enough to begin with) as was shown in (1, 2). So, as the difference between A_2 and A_1 increases, this decreases a (as A_1 is higher, $a = A_2 - A_1$ decreases). Occurrence of the same change to p is prevented as follows: it is likely that at least one of the weights coming to a C neuron is negative. In that case, increasing the difference would involve making the negative weight even more negative, resulting in the neuron joining either A or B (as it no longer fires for the pattern showing the negative weight), thus reducing p. (This is explained further in the supplement)

When the neurons in C remains, we have a more complex case: here, a decreases, but p may also decrease correspondingly; another undesirable scenario is when b decreases to become less than q. Typically sleep tends to drive synaptic weights of the opposite signs, or the weights of same sign but different by some threshold value, away from each other. There are conditions when the difference between weights is below threshold point to cause divergence. In those cases sleep does not improve performance.

Experiments: In our experiments, for majority of the cases, we found C to be empty after sleep, thus making p to become 0. For the instances when this was not a case, the initial values of A_1, A_2, B_1 and B_2 were almost0, i.e., the entire work of classifying the inputs is done by shared input. In such case, the network has no hidden information that sleep could retrieve.

3. Computational Costs of SRC

We can evaluate computational costs of SRC as a combination of (a) number of images passed through the network (both forward and backward passes), and (b) storage costs. We first note that length of sleep is mostly related to the size of the network, not to the size of the training set. Thus, for larger datasets, while more images may be needed during training phase (in the “awake” state) in order for classification accuracy/loss to saturate, during “sleep” this is not necessarily true. Because

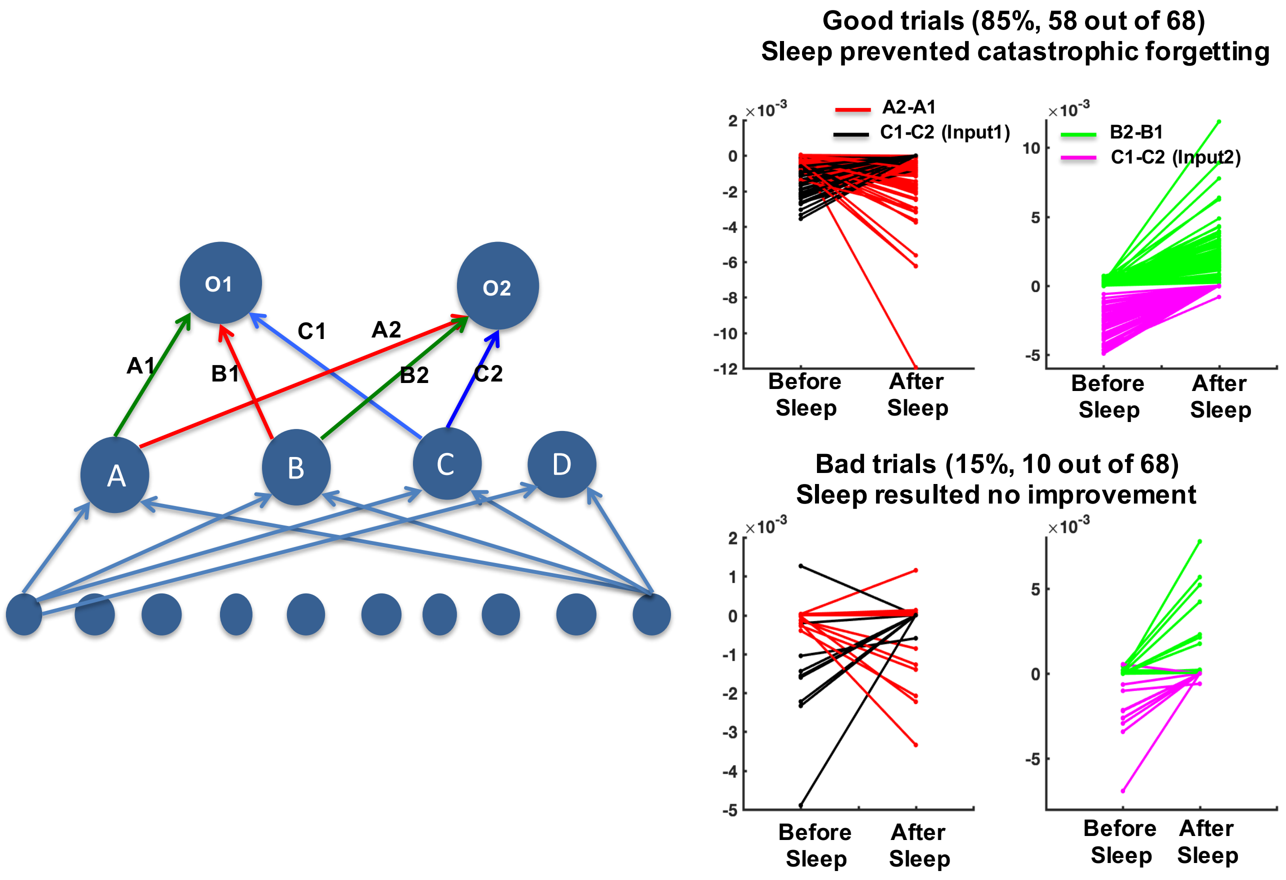

Supplementary Figure 1. Example of the binary vector analysis. In the left graph, we show the structure of the network. A - fires only for input 1. B - fires only for input 2. C - fires for both inputs. D - fires for neither input 1 nor input 2. Green arrows represent desirable connections and red arrows indicate incorrect connections. Blue arrows are mixed depending on the input. The equations on the graphs on the right compare the difference between green and red arrows to the difference between blue arrows (for a given input). Depleting the set of C neurons correspond to giving the differences in the inputs more importance.

of this, the computational cost of sleep is generally similar for networks trained on Imagenet, CIFAR, MNIST, etc (as long as the network size that sleep is applied to is the same, e.g., 2 hidden layers with the same number of units).

As for number of iterations of sleep, we have observed that two parameters dictate how much sleep is necessary to recover performance on old tasks: the magnitude of weight changes (determines how much to increase/decrease weights when an STDP event occurs) and the input firing rate (that is the maximum firing rate for neurons in the first layer during sleep). Parameters can be set so that a very small amount of sleep is needed to recover old task performance. In Supplementary Figure 2, one can see that as the network learns all 5 MNIST tasks, only 500 iterations (and thus 500 feedforward passes through the network) of sleep are needed to reach performance saturation. Each line shows the accuracy as a function of sleep duration on the entire dataset (all 5 tasks). Color indicates the sleep phase (e.g., after training task 1, task 2. etc.)

If sleep requires 500 iterations to ultimately converge, this is equivalent to presenting 500 "noisy" images. In contrast, during training, if we train each class for 2 epochs (as in the case with MNIST), 10,000 images are presented twice, i.e., 20,000 images fed through the network both forwards and backwards for a total of 40,000 passes. SGD is efficient so we do this in batches and we should divide the total number of passes (feedforward + backward) by the batchsize (100). This means the training computational time is $40{,}000/100 = 400$ passes. This is on the same level as what is required during sleep (400-500 passes till convergence). The computational performance of sleep phase can be further improved (e.g., by incorporating the idea of mini-batches, etc.), just as how gradient descent has been heavily optimized.

Lastly, in terms of memory constraints, there are scenarios where SRC reduces storage requirements (as shown with iCaRL) and scenarios where SRC requires more memory (as compared to regularization methods). However, regularization methods may be more computationally intensive, as they require computation of complex matrices, e.g. Fisher information matrix in EWC, or orthogonal projectors in OWM.

4. Effect of Input Type during Sleep

To evaluate the importance of using task averaged input during sleep, we tested SRC for incremental MNIST task with different types of inputs presented during sleep - random input, average input, specific inputs, each presented in 2 conditions (mask/no mask). For random inputs, we build a random uniform vector with the same size as the input space to determine input firing

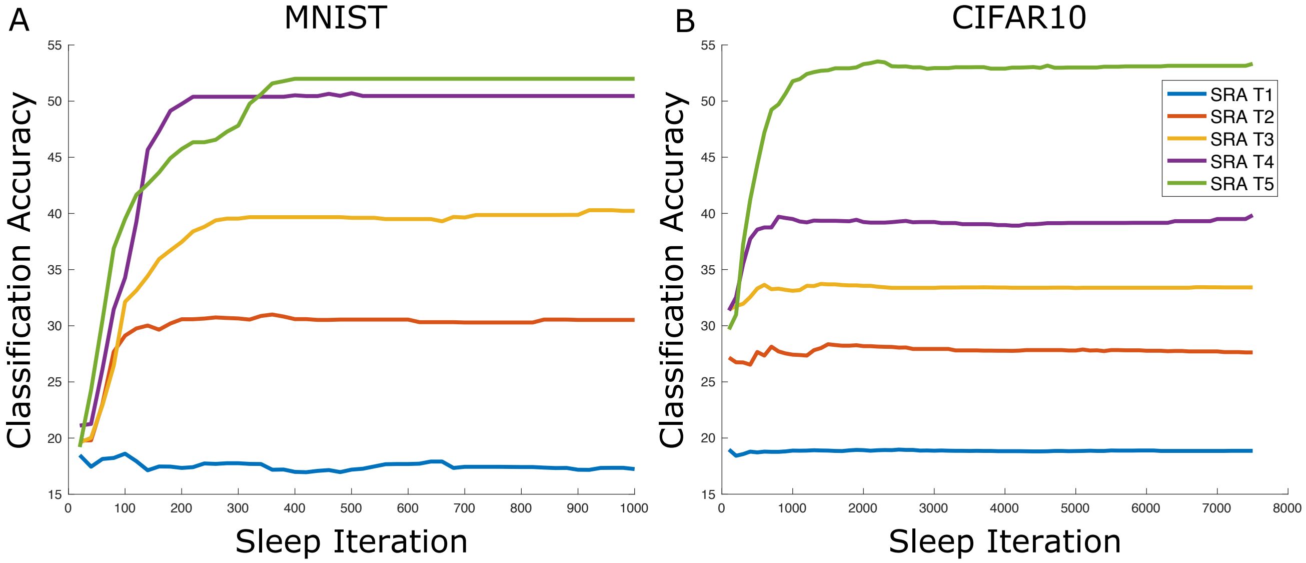

Supplementary Figure 2. Accuracy as a function of sleep iteration for MNIST (left) and CIFAR10 (right) datasets. Each line represents the application of SRC after a specific training phase (e.g., after training T1, T2, etc).

rates of neurons during sleep. For average input, the same input as described in the main paper is used, i.e., the average of all inputs seen so far determines the input firing rates. For specific inputs, we present Poisson distributed spike trains based on specific images (e.g., an image of a 3) during sleep. In the mask condition, during each iteration of sleep we randomly select a 10x10 portion of the input to present during sleep. In the no mask condition, the entire 28x28 vector is used to compute firing rates. Supplementary Figure 3 shows the classification accuracy for each type of input. For specific inputs classification

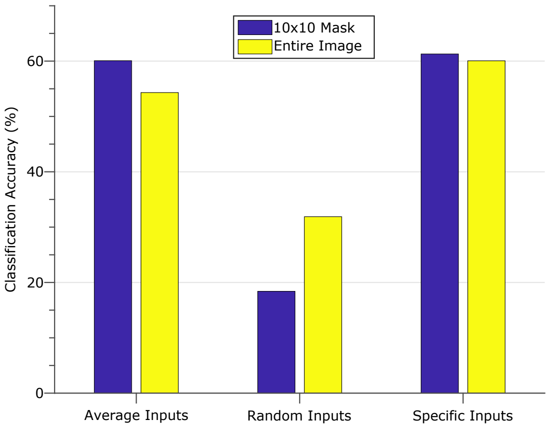

Supplementary Figure 3. Classification accuracy on class-incremental MNIST task with different types of inputs (average, random, specific) with 2 conditions (mask/no mask).

accuracy reaches 60% in both the mask and no mask condition. For average inputs, we see a 10% degradation when the entire image is presented (compared to specific inputs). However, with the mask condition, the degradation between moving from specific to average inputs is much smaller. Finally, looking at random inputs, we observe that when the entire random image is presented, the network is still able to mitigate catastrophic forgetting (task performance is 30%). However, performance is much worse with random inputs, suggesting that task-specific information must be present for the network to optimally recover older tasks.

5. Effect of Task Training Length

Our results suggest that the information about old tasks is not lost in the network after new task training, even if from a classification standpoint the old tasks are not classified correctly. One can expect, however, that as a new task training increases, the network may eventually lose all the old task information. In order to verify this, we measured the classification accuracy before and after sleep in the simplified two task incremental setting for MNIST, Fashion MNIST, CIFAR10, and the crossmodal MNIST task. For MNIST, Fashion MNIST, and CIFAR10, task 1 is the first two classes in the dataset and task 2 is the next two classes. For crossmodal MNIST, task 1 is the entire MNIST task, and task 2 is the entire Fashion MNIST task.

We varied the length of training of task 2 in order to test the hypothesis that longer task 2 training would result in an inability for task 1 to be recovered after sleep since there is less information in the network pertaining to task 1.

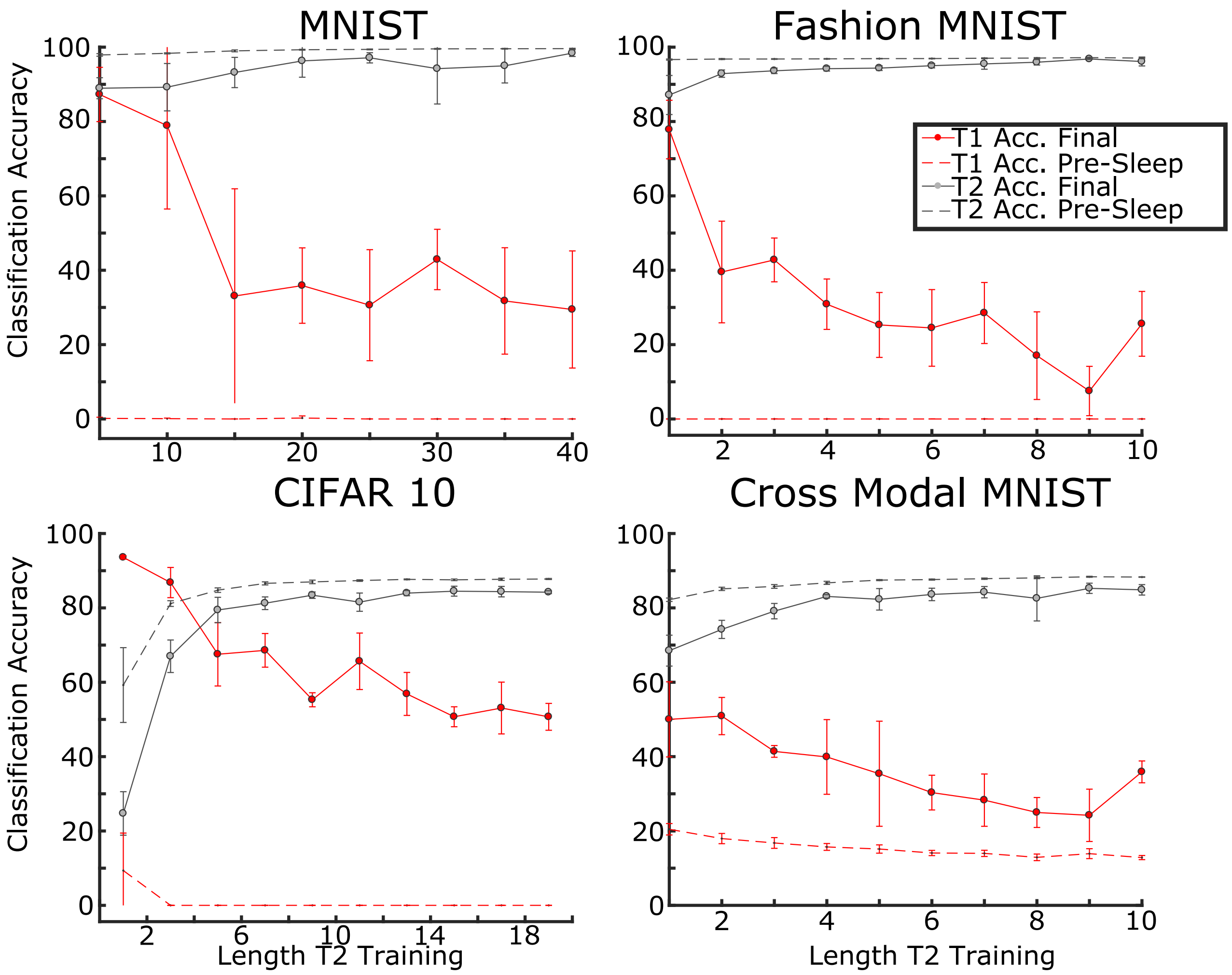

Supplementary Figure 4. Classification accuracy as a function of length of task 2 training (in epochs) for 2 task MNIST, Fashion MNIST, and CIFAR 10 as well as the Cross Modal MNIST task. Solid red - T1 accuracy after sleep, dashed red - T1 accuracy before sleep (after T2 training), dashed gray - T2 accuracy before sleep (after T2 training), and solid gray - T2 accuracy after sleep.

Supplementary Figure 4 shows classification accuracy on task 1 and task 2 before and after sleep, for all 4 datasets as a function of the length of task 2 training (in epochs). In all cases, T1 is mostly forgotten after T2 training (red dashed line), except in the case of Cross Modal MNIST, where T1 accuracy is around 20%. With light task 2 training, task 1 classification accuracy is recovered significantly. However, as we increase the length of task 2 training, task 1 recovery decreases. Nevertheless, it never becomes completely forgotten after sleep (solid red line is always above dashed red). Note that the reverse is true for T2. For light T2 training, T2 becomes more degraded after sleep. With longer T2 training, T2 performance remains unchanged after sleep (dashed gray line = solid gray line).

These results also suggest that possibly effective training strategy would be to interleave multiple episodes of new task training and sleep. Indeed, this would well match biology, when new procedural task training in human brain happens slowly and involves multiple training sessions and multiple episodes of sleep between them.

6. Effect of SRC on Single Task Performance

From neuroscience standpoint the best studied effect of sleep on memory is an improvement of a single task performance after sleep. E.g., in experiments with new skill learning, comparing task performance following episode of training + sleep vs. training with no sleep reveals memory improvement when sleep is included. In fact, recent studies revealed an inverse association between learning performance and gains in procedural skill, i.e., good learners exhibited smaller performance gains after sleep than poor learners (3).

To test if SRC can provide similar performance benefits for a single task learning and also show similar dependence on

pre-sleep performance, we used the entire MNIST dataset and trained it in 4 conditions, that were different by the length of training. SRC was implemented after training in all 4 cases and we compared performance before vs. after sleep.

Supplementary Figure 5 shows the classifcation accuracy on the entire MNIST dataset before/after sleep in 4 training conditions. When training is short, i.e., pre-sleep performance is low, we found a very significant 20 percentage point increase in classification accuracy after SRC. When training is long, i.e., pre-sleep performance is high, we see a 5 percentage point improvement. Finally, for very long training, we observed small reduction in performance after SRC. The last may be related to the reported SRC role in increasing generalization (4). Indeed, requirements for robustness/generalization and classification accuracy are conflicting, so increase in generalization may explain observed reduction of accuracy after SRC even on recent tasks. These results suggest that replay during SRC can indeed capture some important elements of replay in the biological brain.

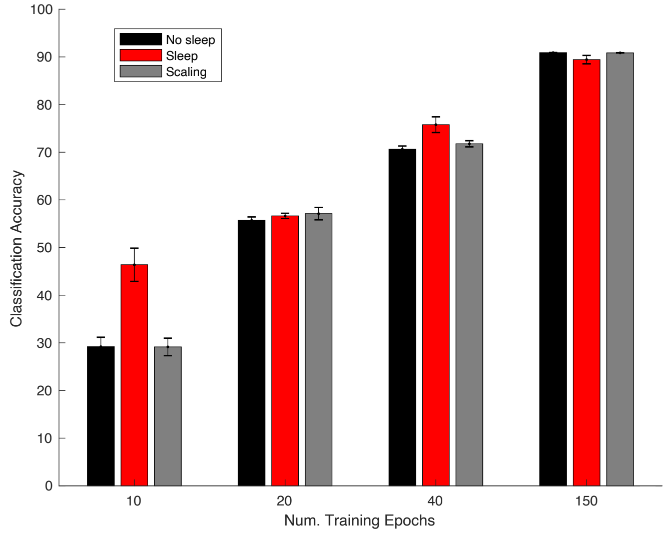

Supplementary Figure 5. Classification accuracy on the MNIST dataset before (black) and after (red) sleep when trained for 10, 20, or 40, 150 epochs. Scaling of top 2.5% of weights by 1% shown in grey.

7. Implementation of Other Methods

Elastic Weight Consolidation. Elastic weight consolidation (5) seeks to reduce the plasticity of weights that are deemed important for previously learned tasks, while utilizing less important weights to learn and represent the new task. Any updates to weights deemed important for previously learned tasks are penalized in the loss function, thus making it harder for any new training to impact the performance of old tasks. More formally, this is written as a regularization term added onto the loss function

$$L(\theta) = L_B(\theta) + \sum_i \frac{\lambda}{2} F_i(\theta_i - \theta^*_{A,i}).$$

Here $L_B(\theta)$ represents the normal loss function evaluated on the new training set B. The regularization term computes the product of the Fisher information matrix of parameter i with the difference between the proposed weight update θ_i and the original parameter when trained on an earlier dataset $\theta_{A,i}$. In this way, weight updates to parameters deemed important for task A are penalized. The λ parameter determines how plastic the network should be. A high value for λ suggests that weight updates should only occur when they are vital to achieving a high performance on the new task B. For our purposes, we performed a parameter sweep over λ to find the best value of λ for each specific task.

In this work, we implemented elastic weight consolidation (EWC) as a benchmark to compare against the sleep replay algorithm. In the original work (5), EWC was tested on the MNIST permutation task as well as a series of reinforcement learning games. In other works, it has been noted that EWC does not achieve optimal results on incremental learning tasks (when the classes in the dataset are learned incrementally) (6, 7). In this work, we corroborate these results. Mainly, EWC fails to perform continual learning on the incremental learning tasks. However, on the Multi-modal MNIST task where the network must learn both the Fashion MNIST and MNIST datasets, EWC outperforms SRC. These results suggests that different methods can excel in certain areas of continual learning but fail in others and that combinations of different methods may provide an optimal solution that can be explored in future studies.

Synaptic Intelligence. The Synaptic Intelligence approach (SI) (8) is similar to EWC with a slightly different regularization term. To penalize updates to important parameters, the SI method uses the following loss function:

$$L'_\mu = L_\mu + c\sum_i \Omega^\mu_i(\theta'_i - \theta_i)^2.$$

This regularization term is similar to EWC, where Ω_i^μ keeps track of how important an indidual parameter θ_i is to previously learned tasks. The parameter c is similar to the λ parameter in EWC. We performed a parameter search over c to find the best value for each of the tasks tested in the paper. Given the similarities of the two approaches, we observed similar performances on the tasks tested in the paper between EWC and SI. Mostly, SI fails to perform class incremental learning but performs well on the Multi-modal MNIST task.

Orthogonal Weight Modification. The orthogonal weight modification (OWM) approach (9) takes a different approach by trying to force weight updates in a direction that is orthogonal to the subspace spanned by all previously learned inputs. First, a projector P is constructed to find the direction orthogonal to the input space: $P = I - A(A^TA + \alpha I)^{-1}A$, where A consists of all previously trained inputs as columns. Weight modifications are then done by $\Delta W = \kappa P \Delta W^{BP}$. We used α values of $0.9 * (0.001^l)$, 0.1^l, and 0.6 in each of the 3 hidden layers. Here, l represents the progress made through the training of the current task. For CIFAR-10, we used $0.9 * (0.0001^l)$, 0.1^l, and 0.006 as the α parameters. The authors in the original paper use an iterative method to construct P so not all inputs must remain stored. In the tasks tested in the paper, OWM performs significantly better than the other two regularization methods: EWC and SI.

Additional analyses revealed that OWM may take longer to converge than standard methods. An ideal ANN may converge on MNIST after around 2 epochs/task. However, this was not true for OWM. Table 1 in the manuscript shows OWM performance when trained with 10 epochs/task (the same as for all other methods) on MNIST, Fashion MNIST and CIFAR10. We chose this training time as it was sufficient to converge for all the methods, including those with slow convergence rate. In Supplementary Figure 6, we present data over a range of training duration for OWM. The results with longer training are in line with other implementations of OWM except for the case of CIFAR-10 (9, 10). We hypothesize that this disparity occurs because our study only applies OWM to the fully connected layers, not in an end-to-end fashion, as done in (9). Since our extracted features for CIFAR10 images were based on Tiny Imagenet, they are likely not maximally informative. If we use extracted features from a network trained on CIFAR10 instead of Tiny Imagenet, we could improve OWM performance on CIFAR10 (when trained sequentially) from 34% to 42%. This suggests that OWM may work better in scenarios where the feature extractor can also be fine-tuned on the dataset.

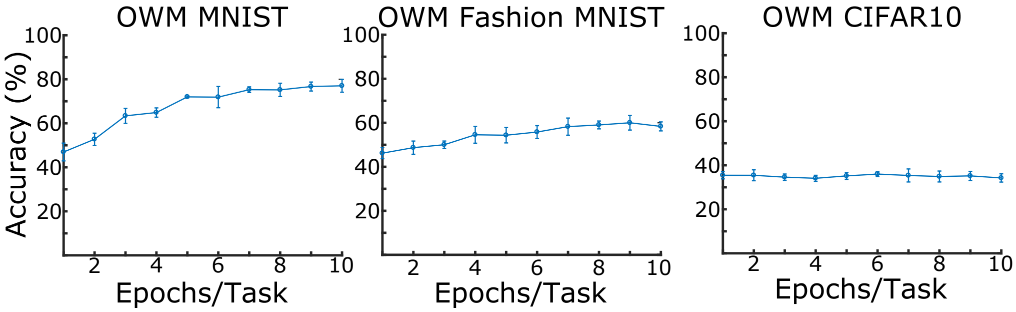

Supplementary Figure 6. Analysis of OWM performance as a function of number of epochs per task on MNIST (left), Fashion MNIST (middle) and CIFAR10 (right) datasets.

Discussion of Regularization Approaches. Table 1 in the main paper illustrates that the first two regularization (SI and EWC) approaches fail to prevent catastrophic forgetting in the incremental tasks. However, on the Multi-modal MNIST task, these regularization approaches come close to reaching the ideal accuracy. Here, we briefly discuss why these methods may succeed in some domains and fail in others. In the paper outlining EWC, the authors tested the algorithm on the MNIST permutation task and reinforcement learning tasks (5). For SI, the algorithm was tested on the MNIST permutation task as well as the split MNIST and CIFAR tasks (8).

The MNIST permutation task defines a single task as one representation of the input space. During subsequent tasks, this representation is permuted (so that pixel i of all images now appears in a new location in the image). During incremental learning, the network must be able to distinguish all 10 digits in previously learned permutations, while also learning the newest permutation. Since a lot of the information in MNIST images is present in the center of the image, we hypothesize that when a new permutation of the images is created, the pertinent information is likely moved to distinct regions of the image. These regions have a relatively small amount of overlap with the old permutations, so when EWC or SI are applied, important weights connecting from the center of the image may be preserved, while the weights that connect from the new important regions of the image are updated.

For split MNIST/CIFAR tasks, the classes in the dataset are divided into subsets (i.e. pairs of digits or images), and the network learns to distinguish each image in the pair. During task 1, the network may learn to distinguish 0 and 1. Then, during task 2, the network may learn to distinguish 2 and 3, utilizing the same two output neurons as were used for task 1. In this scenario, regularization approaches may work well by allocating distinct pathways through the network for each binary classification task learned. Thus, when task 2 is learned, old pathway through the network is preserved for task 1, and a new pathway is created to represent task 2. In incremental learning settings, activity to certain output neurons is cut off during new task training, as is the case with the incremental tasks tested in the paper. Thus, when training on a new task (e.g., digits 2 and 3), output neurons representing old memories (e.g., digits 0 and 1) should not receive any activity to achieve a suitable

loss on the new task. We hypothesize that the regularization term added by EWC and SI is insufficient to maintain activity to these output neurons representing older classes.

Nevertheless, we found that EWC and SI work well for the multi-modal MNIST task (where the network must learn MNIST and Fashion MNIST incrementally). In this task, during both phases of learning, all output neurons remain active. Additionally, the MNIST and Fashion MNIST datasets may maintain information in distinct parts of the image. Therefore, regularization approaches may consolidate the two tasks into the network, solely by preserving weights that are significantly important for the old task and using the relatively less important weights to represent the new task.

Incremental Classifier and Representation Learning (iCaRL). Here, we describe our implementation of the iCaRL method and differences with the original paper (11). The main idea of the iCaRL algorithm is to utilize a fixed memory capacity (capacity K, where K represents number of examples stored from previous classes) in an efficient way to prevent catastrophic forgetting. Specifically, it selects which K examples to store from previous classes ($m = \frac{K}{t}$ images per class, where t is the number of classes seen so far) by adding examples to the memory bank which cause the average feature vector over all exemplars (m images in the memory bank) to approximate the average feature vector over all training examples (all images of the relevant class). These images are sorted in the exemplar set based on how well they approximate the average feature vector over all training examples. When a new class is learned, the exemplar set for a certain class is reduced (now $m = \frac{K}{t+1}$) by removing the least informative (of the average feature vector) examples.

In addition to introducing an efficient exemplar management, the learning rule and classification scheme are modified in the original iCaRL implementation (11). Specifically, the learning rule incorporates both soft-target distillation over examples from previous tasks as well as cross-entropy loss from the newest task. The labels for the new images are binary one-hot encoded vectors with the correct classifications. However, the labels for the old images are computed by passing these images through the network and storing the output from the network. In addition, the classification scheme is changed to use the Nearest Means of Exemplar classification strategy, where test inputs are fed through the network and their representation in the last hidden layer (before the classification layer) is used to compute the classification for that test example. Its label is determined by finding the nearest-class-mean using the exemplar set and all class labels which have been learned so far. In our implementation of iCaRL, we used the Nearest Means of Exemplar classifier as well as soft-target distillation.

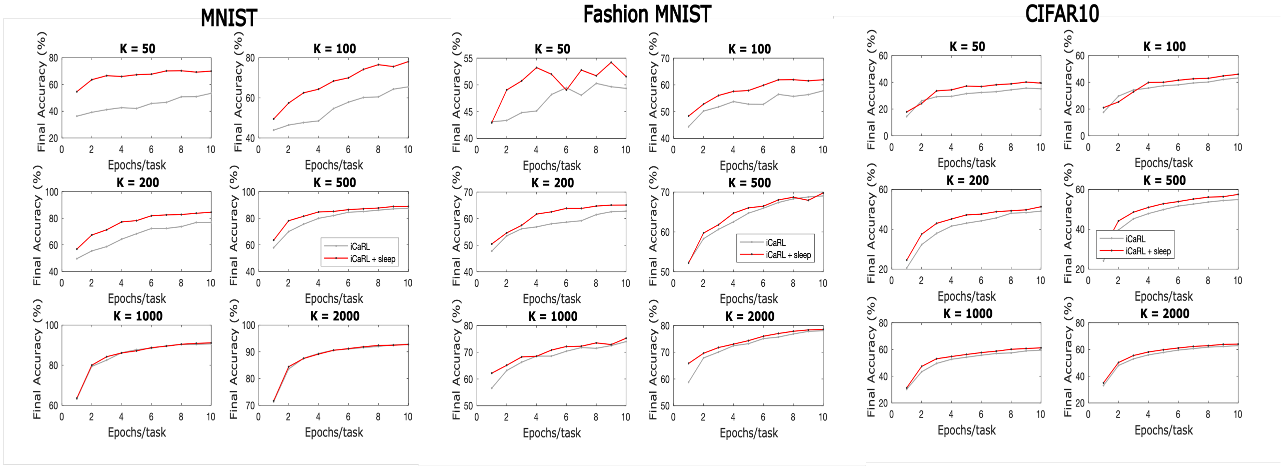

Supplementary Figure 7. Analysis of iCaRL performance with (red) and without (gray) SRC as a function of number of epochs per task on MNIST (left), Fashion MNIST (middle) and CIFAR10 (right) datasets.

As with OWM, we found that iCaRL can benefit from longer training times (see Supplementary Figure 7). We, however, showed that even with longer training times, SRC can still improve upon iCaRL performance, especially for lower values of K. Furthermore, SRC can reduce the training time needed to achieve maximal performance of iCaRL (Supplementary Figure 7). We characterize the savings in the training time by the difference in epochs/task when iCaRL + SRC achieves the same accuracy as iCaRL alone after 10 training epochs. These results are reported in the main manuscript.

8. Implementation of both Training and Sleep Replay within SNN

Sleep replay algorithm presented in this study was implemented for artificial neural networks. In addition, we tested another implementation when both training and sleep were implemented using spiking neural networks (SNNs). SNNs recently received attention from the neuromorphic hardware community for their ability to perform power-efficient computing (12), particularly during inference phase (13–15). However, SNNs are still inferior to ANNs on complex data training (16). Nevertheless, the question is raised of whether or not the entire wake-sleep pipeline could be performed within a spiking network architecture as well as what implications this may have for neuroscience and computer science.

To address this question, we implemented both awake training and sleep replay withing SNN architecture. We performed "awake" sequential training on MNIST data in an SNN via backpropagation (17). This method works by approximating the

discontinuity in a neuron's non-linear spiking function so as to make it differentiable when performing backpropagation. Our goals were (a) to verify that catastrophic forgetting occurs in an SNN trained through backpropagation; (b) to test the effect of SRC in overcoming catastrophic forgetting.

Phase	T1 performance	T2 performance
T1 awake training	99.6	0.6
Sleep	84.8	2.9
T2 awake training	0	85.9
Sleep	71.8	51.3

Supplementary Table 1. Catastrophic forgetting is observed when sequential training is performed in an SNN architecture. SRC is able to produce more balanced classification accuracy on both tasks (T1 = digits 0-1, T2 = digits 2-3 in MNIST dataset). Each value represents the average of 5 trials.

Supplementary Table 1 illustrates the classification accuracy for two task incremental training on the MNIST dataset (T1 = digits 0-1, T2 = digits 2-3). The SNN trained here had the same number of neurons in each layer as the ANNs used in the main paper (2 hidden layers with 1200 neurons each). After training on T1, SNN was able to classify images in T1 with 99% classification accuracy (averaged over 5 trials). After training on T2, catastrophic forgetting occurred, as classification accuracy on T1 dropped to 0%. This illustrates that catastrophic forgetting is not unique to ANNs but can occur in SNNs due to the interference between the tasks. (Indeed, our recent studies suggest that catastrophic forgetting can occur in a spiking network even with biologically realistic local learning rules (18, 19) when tasks with a high amount of overlap are learned incrementally.) After sleep, performance on T1 was mostly recovered while performance on T2 was partially reduced. Overall, this experiment demonstrates that catastrophic forgetting does occur in SNNs and sleep can reduce the impact of catastrophic interference.

9. Analysis of Sleep Replay

To evaluate replay of the old and new tasks during sleep, we compared the neurons that spiked frequently during sleep with the average activation in the ANN for each specific image (Supplementary Figure 8). When an image was presented to the ANN (before SRC is applied), we recorded the activation vectors in each hidden layer a_{1,i_j}, a_{2,i_j} for hidden layers 1 and 2, respectively, for each image i of class j. For two tasks, we recorded values for a_{1,i_0} through a_{1,i_3} and a_{2,i_0} through a_{2,i_3} to store activation vectors for each class of digits learned, 0 through 3. Then, we tested for the overlap between neurons that had non-zero activation values in ANN (before SRC) for a specific input and neurons which had non-zero firing rates during subsequent sleep (during SRC). This serves as a proxy to measure the extent to which a specific input is replayed during sleep. As a control group, we performed the same calculations but using ANN activation vectors obtained in response to an image created with uniformly-distributed random noise.

Supplementary Figure 8A shows the average amount of reactivation for each digit in each hidden layer, normalized by the reactivation of the control group. In layer 1, for each digit, we observed reactivation values that were greater than reactivation for a random input. Most notably, digit 1 (from the old task) seems to be reactivated the most, followed by digits 2 and 0. Analysing the confusion matrices before and after sleep for this specific network (Fig 8B), we observed that the classification accuracy for digit 1 was particularly improved after sleep, and when a network predicted an image incorrectly, it often misclassified it as digit 1. This suggests that the digit-specific reactivation correlates well with the network classification performance and further steps to improve reactivation during sleep may lead to further SRC performance improvements. In Layer 2 (Fig 8A, right), we observed that all digits were reactivated greater than expected for a random input image. However, the ratio of reactivation for each digit was about equal. Overall, these results suggest that previously learned tasks are replayed during sleep phase and amount of replay positively correlates with classification performance on the old digits that otherwise would have been forgotten.

In addition to reactivation, we also measured the effect of sleep replay on the representation of distinct digits in the network. Work by (20) shows that during training, representations of distinct image classes can become more dissimilar and sparser. We perform a similar analysis here by plotting the activation of a group of 40 randomly selected neurons before and after sleep, when presented with each of the 10 MNIST digits (Fig 9). Before sleep, the representation of all digits is very similar (with the exception of digits 8 and 9, which were more recently learned). After sleep, every digit has a unique representational code, along with much sparser activity.

Supplementary References

1. Y Wei, GP Krishnan, M Bazhenov, Synaptic mechanisms of memory consolidation during sleep slow oscillations. *J. Neurosci.* **36**, 4231–4247 (2016).
2. OC González, Y Sokolov, G Krishnan, M Bazhenov, Can sleep protect memories from catastrophic forgetting? *BioRxiv*, 569038 (2019).
3. FH Rångtell, et al., Learning performance is linked to procedural memory consolidation across both sleep and wakefulness. *Sci. reports* **7**, 1–8 (2017).
4. T Tadros, G Krishnan, R Ramyaa, M Bazhenov, Biologically inspired sleep algorithm for increased generalization and adversarial robustness in deep neural networks in *International Conference on Learning Representations.* (2019).
5. J Kirkpatrick, et al., Overcoming catastrophic forgetting in neural networks. *Proc. national academy sciences* **114**, 3521–3526 (2017).
6. GM van de Ven, AS Tolias, Generative replay with feedback connections as a general strategy for continual learning. *arXiv preprint arXiv:1809.10635* (2018).
7. GM van de Ven, HT Siegelmann, AS Tolias, Brain-inspired replay for continual learning with artificial neural networks. *Nat. communications* **11**, 1–14 (2020).
8. F Zenke, B Poole, S Ganguli, Continual learning through synaptic intelligence in *Proceedings of the 34th International Conference on Machine Learning-Volume 70.* (JMLR. org), pp. 3987–3995 (2017).
9. G Zeng, Y Chen, B Cui, S Yu, Continual learning of context-dependent processing in neural networks. *Nat. Mach. Intell.* **1**, 364–372 (2019).
10. TC Kao, K Jensen, G van de Ven, A Bernacchia, G Hennequin, Natural continual learning: success is a journey, not (just) a destination. *Adv. Neural Inf. Process. Syst.* **34** (2021).

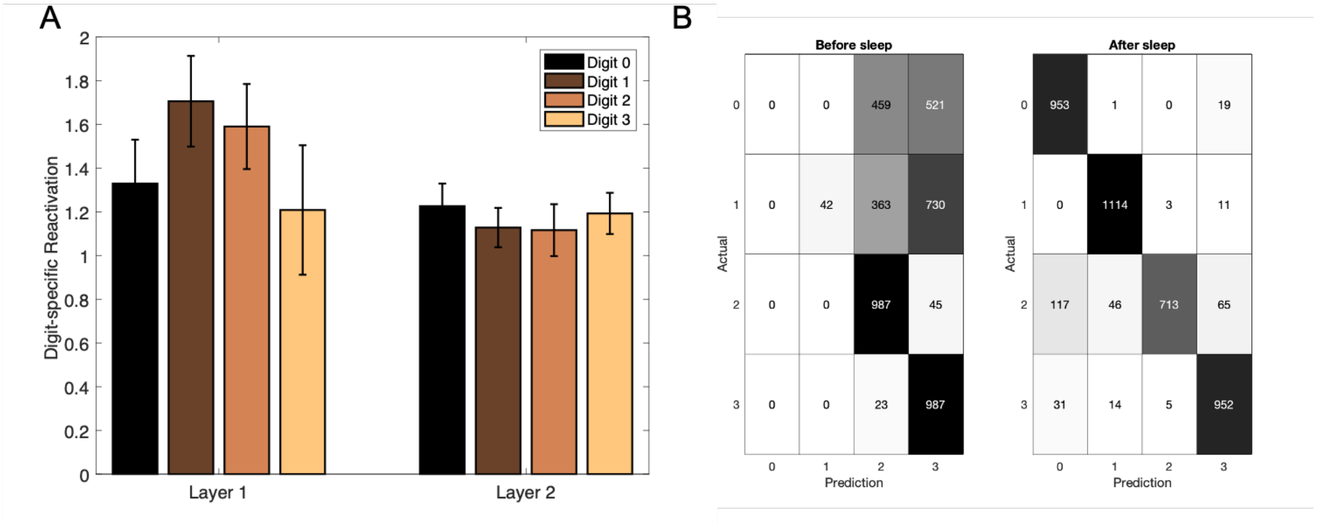

Supplementary Figure 8. During sleep, digit-specific activations are replayed more often than would be expected by chance. A) Digit-specific reactivation in layer 1 (left) and layer 2 (right) for each digit. Each value is normalized by a control group so values greater than 1 indicate there is more reactivation than would be expected for a randomly generated image. B) Confusion matrices before (left) and after (right) sleep for the two tasks.

11. SA Rebuffi, A Kolesnikov, G Sperl, CH Lampert, icarl: Incremental classifier and representation learning in *Proceedings of the IEEE conference on Computer Vision and Pattern Recognition.* pp. 2001–2010 (2017).
12. S Ghosh-Dastidar, H Adeli, Spiking neural networks. *Int. journal neural systems* **19**, 295–308 (2009).
13. PU Diehl, et al., Fast-classifying, high-accuracy spiking deep networks through weight and threshold balancing in *2015 International joint conference on neural networks (IJCNN).* (ieee), pp. 1–8 (2015).
14. L Zhang, S Zhou, T Zhi, Z Du, Y Chen, Tdsnn: From deep neural networks to deep spike neural networks with temporal-coding in *Proceedings of the AAAI Conference on Artificial Intelligence.* Vol. 33, pp. 1319–1326 (2019).
15. S Davidson, SB Furber, Comparison of artificial and spiking neural networks on digital hardware. *Front. Neurosci.* **15** (2021).
16. Y Wu, et al., Direct training for spiking neural networks: Faster, larger, better in *Proceedings of the AAAI Conference on Artificial Intelligence.* Vol. 33, pp. 1311–1318 (2019).
17. A Yanguas-Gil, Coarse scale representation of spiking neural networks: backpropagation through spikes and application to neuromorphic hardware in *International Conference on Neuromorphic Systems 2020.* pp. 1–7 (2020).
18. OC González, Y Sokolov, GP Krishnan, JE Delanois, M Bazhenov, Can sleep protect memories from catastrophic forgetting? *Elife* **9**, e51005 (2020).
19. R Golden, JE Delanois, P Sanda, M Bazhenov, Sleep prevents catastrophic forgetting in spiking neural networks by forming joint synaptic weight representations. *bioRxiv*, 688622 (2020).
20. JL McClelland, BL McNaughton, RC O'Reilly, Why there are complementary learning systems in the hippocampus and neocortex: insights from the successes and failures of connectionist models of learning and memory. *Psychol. review* **102**, 419 (1995).

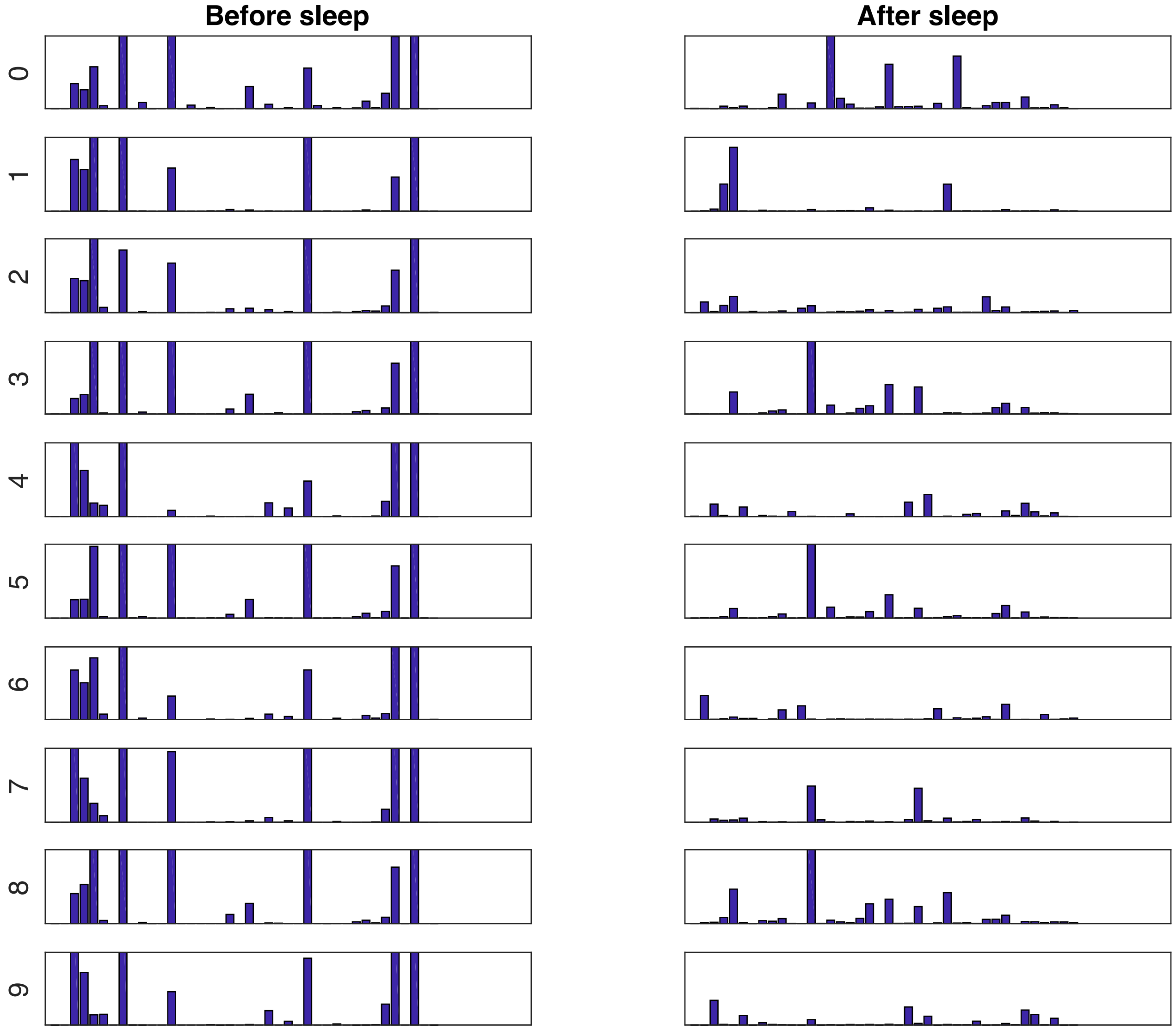

Supplementary Figure 9. Average activation of 40 randomly selected neurons before (left) and after sleep (right) when presented with digits from each class. Overall, less neurons respond to each digit suggesting a sparser representation.
